# Increasing Robustness of Brain–Computer Interfaces Through Automatic Detection and Removal of Corrupted Input Signals

**DOI:** 10.3389/fnins.2022.858377

**Published:** 2022-04-28

**Authors:** Jordan L. Vasko, Laura Aume, Sanjay Tamrakar, Samuel C. IV Colachis, Collin F. Dunlap, Adam Rich, Eric C. Meyers, David Gabrieli, David A. Friedenberg

**Affiliations:** ^1^Battelle Memorial Institute, Columbus, OH, United States; ^2^Department of Biomedical Engineering, The Ohio State University, Columbus, OH, United States

**Keywords:** brain–machine (computer interface), neuroprosthetic, deep learning – artificial neural network, intracortical array, statistical process control

## Abstract

For brain–computer interfaces (BCIs) to be viable for long-term daily usage, they must be able to quickly identify and adapt to signal disruptions. Furthermore, the detection and mitigation steps need to occur automatically and without the need for user intervention while also being computationally tractable for the low-power hardware that will be used in a deployed BCI system. Here, we focus on disruptions that are likely to occur during chronic use that cause some recording channels to fail but leave the remaining channels unaffected. In these cases, the algorithm that translates recorded neural activity into actions, the neural decoder, should seamlessly identify and adjust to the altered neural signals with minimal inconvenience to the user. First, we introduce an adapted statistical process control (SPC) method that automatically identifies disrupted channels so that both decoding algorithms can be adjusted, and technicians can be alerted. Next, after identifying corrupted channels, we demonstrate the automated and rapid removal of channels from a neural network decoder using a masking approach that does not change the decoding architecture, making it amenable for transfer learning. Finally, using transfer and unsupervised learning techniques, we update the model weights to adjust for the corrupted channels without requiring the user to collect additional calibration data. We demonstrate with both real and simulated neural data that our approach can maintain high-performance while simultaneously minimizing computation time and data storage requirements. This framework is invisible to the user but can dramatically increase BCI robustness and usability.

## Introduction

Despite significant progress in intracortical brain computer interface (BCI) technology, there are few examples of practical use in home environments (Collinger et al., 2013b; Murphy et al., 2016; Oxley et al., 2020; Weiss et al., 2020; Cajigas et al., 2021). One obstacle to translation is that chronic BCI systems are likely to encounter signal disruptions due to biological, material, and mechanical issues that can corrupt the neural data (Barrese et al., 2013; Dunlap et al., 2020). While some disruptions can be catastrophic and cause complete signal loss, disruptions often only affect a subset of BCI recording channels (Colachis et al., 2021). When this happens performance may be fully or partially recovered by removing the affected channels and using only the unaffected channels. Ideally, an algorithm could automatically detect these disruptions, flag them for possible repair, and then reoptimize the decoder to maximize performance using the unaffected channels. For deployed home use systems, these steps should occur automatically and be invisible to the user. Furthermore, the adjustments should be computationally efficient to minimize recalibration time and data storage needs, as they will be run on mobile systems with limited, battery-powered hardware.

To our knowledge, no fully automated method exists to detect the myriad possible signal disruptions that can occur in intracortical BCI systems. To address this problem, we have adapted statistical process control (SPC) methodology to monitor BCI data. SPC is a quality-control framework for inferring changes to a process over time by specifying criteria to identify samples that deviate from the typical behavior of the signal, referred to as “out-of-control” samples (Western Electric Company, 1956). SPC methodology is typically applied to manufacturing processes, but with slight modifications lends itself very well to chronic tracking of neural data. Here we demonstrate how it can be used to detect disrupted signals in BCI systems. Notably this approach is independent of the neural decoder and applicable for a broad range of BCI applications.

In controlled laboratory work, a decoder such as a support vector machine (SVM), linear model, or Kalman filter is typically calibrated *de novo* at the beginning of each session using new calibration data collected from the user to account for expected daily variability in the recorded signals (Hochberg et al., 2012; Collinger et al., 2013c; Bouton et al., 2016; Sharma et al., 2016; Ajiboye et al., 2017; Friedenberg et al., 2017; Colachis et al., 2018). In this situation, if corrupted channels have been identified, they can be excluded from the daily recalibrated decoder. Even without exclusion of corrupted channels, the daily recalibration of decoders should minimize the importance of channels whose signals have minimal association with the user’s intended action. Thus, typically no additional algorithmic handling of signal disruptions is necessary with daily recalibration. However, the collection of labeled calibration data presents a significant time investment for BCI users, and surveys of potential users suggest that many would not be willing to invest time to retrain the decoder every day in a deployed system (Huggins et al., 2011, 2015; Collinger et al., 2013a). Thus, several groups have developed neural decoding approaches that are less reliant on daily recalibration.

Deep learning models trained using multiple sessions of historical data can maintain high performance while eliminating the need to retrain the decoder from scratch each session (Sussillo et al., 2016; Schwemmer et al., 2018; Skomrock et al., 2018; Rodrigues et al., 2019). Additionally, decoders trained previously can be updated using unsupervised methods that do not require the user to collect daily calibration data (Jarosiewicz et al., 2015; Kao et al., 2017; Schwemmer et al., 2018; Degenhart et al., 2020). These unsupervised methods update decoder weights based on general use data, allowing the decoder to adapt to changes in neural dynamics over time. Importantly, all these methods are robust to relatively small daily variations but will not necessarily accommodate abrupt and drastic disruptions to the neural data that can happen due to physical damage to the sensor or recording equipment, which can cause catastrophic loss on the affected channels.

Thus, groups have developed decoders robust to corrupted channels without needing to retrain the decoder completely. Sussillo et al. (2016) demonstrated a high-performing multiplicative recurrent network (MRNN) that tolerated the zeroing of three to five of the most informative electrodes with only moderate performance decrements compared to when no electrodes were lost. The algorithm could adapt to signal perturbations due to data augmentation from perturbed spike counts and incorporation of past neural activity into the MRNN. Kao et al. (2017) similarly demonstrated high decoding performance using a hysteresis neural dynamical filter (HDNF). Performance was similar to the case of no damage when up through approximately 10 of the most informative electrodes were removed for the 96-electrode system, and up through approximately 50 of the electrodes for the 192-electrode system. The robustness of the model to lost electrodes was accredited to its “memory” of previous states when all electrodes were available. Although these models show some robustness to certain types of channel loss, they lack the automated flagging of corrupted channels which may be repairable, and they do not remove the corrupted channels from the model. Furthermore, these methods only test model robustness by zeroing channel input, thus simulating “dead” channels. It is unclear whether these methods would be robust to non-zero corrupted input signals, such as those created by floating or shorted channels.

Additional approaches have been implemented in machine learning literature to increase robustness of deep neural network algorithms. One commonly used method is dropout, in which a randomly selected subset of weights between layers is zeroed. This approach is similar to the data augmentation approach implemented by Sussillo et al. (2016) during training of their MRNN, and approximates the simulated lost connections introduced by both Sussillo et al. (2016) and Kao et al. (2017) during decoder evaluation. Another such method is mixup (Zhang H. et al., 2018), which is believed to reduce overfitting by the model by augmenting the training data with linear combinations of the existing training examples. Dropout and mixup were designed to reduce overfitting by the model during training by preventing overreliance on any particular input feature. By reducing overreliance on individual input channels, these methods should also increase robustness to damage on a subset of channels. To our knowledge, mixup has not yet been applied to neurological signals. Both methods will be applied in the models explored below.

**FIGURE 1:**
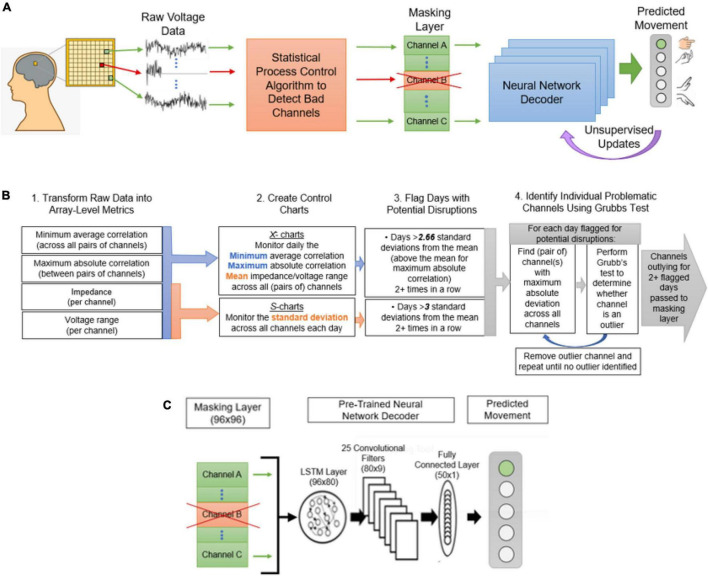
The proposed decoding framework for handling channel disruptions. **(A)** Raw voltage data and impedance measurements recorded from the implanted electrode array will be delivered to the statistical process control algorithm, where channels with disruptions will be identified. The discovery of disruptions will cause the input from the affected channels to be masked, or zeroed, before being sent to the decoder. This will also trigger an unsupervised update to readjust the weights to the missing input. **(B)** The statistical process control algorithm to detect disrupted channels is described. The four steps in this process are (1) transforming raw data into four array-level metrics useful for signal monitoring, (2) creating control charts for each of the metrics, (3) using the control charts to flag sessions with potential disruptions, and (4) performing Grubb’s test to determine outlying channels on the flagged sessions. **(C)** The decoder framework, including a masking layer, an LSTM with 80 hidden units, 25 convolutional filters, a fully connected layer, and finally a 5-unit layer corresponding to the output.

In the following, we introduce a novel, automated approach for dealing with corrupted channels by (1) automatically identifying problematic channels by adapting established statistical process control (SPC) techniques, (2) inserting a masking layer in neural network decoder architectures to remove the problematic channels without retraining from scratch and, (3) unsupervised updating to reassign the weights of the remaining channels without requiring the user to explicitly recalibrate. Figure 1 presents an overview of the proposed system for decoding neural signals in the presence of damaged channels, including the interaction with the modified SPC process, channel masking layer, and unsupervised updates. Using SPC, key channel health metrics like impedance and channel correlations are monitored over time, yielding baselines and tolerance bounds for normal operating behavior. Channels within the tolerance bounds pass through the model unaltered. If any channel metrics exceed the tolerance bounds, the identified channels are determined disrupted and then removed in the channel masking layer so they cannot influence subsequent decoding layers. Importantly, the channel layer removes channels without changing the model architecture—this enables methods such as transfer learning and fine-tuning to adapt the decoder in a computationally efficient manner. Unsupervised updating can then continually improve the model without placing any additional burden on the user. While we demonstrate our approach using a specific set of SPC parameters and a specific neural network architecture, the approach is highly generalizable. SPC methods are completely independent of the neural decoder and can be applied wherever there is sufficient historical data to establish a baseline and assess variability. Additionally, the masking and unsupervised updating approaches are flexible and agnostic to the neural network architecture. In the following, we detail our approach and demonstrate these methods using clinical data collected over a 5-year study with an implanted Utah array BCI as well as simulated neural signal disruptions.

## Materials and Methods

The following sections describe the data collected via a motor imagery task presented to a human participant with an implanted microelectrode array (MEA), the modified statistical process control approach developed to identify disruptions in the signal recorded from the MEA, and the models implemented to predict the participant’s intended movements. A neural network model incorporating the proposed channel masking layer and unsupervised updates was compared against several control models which are also described. All decoding and SPC analyses were performed offline in Python 3.6 (Van Rossum and Drake, 2009). Mixed effects modeling and analysis was performed offline in R 3.6.2 (R Core Team, 2021).

### Datasets

This investigational clinical trial was approved by United States Food and Drug Administration (FDA) under an Investigational Device Exemption (IDE) and The Ohio State University Wexner Medical Center Institution Review Board (Columbus, OH, United States) and registered on ClinicalTrials.gov (Identifier NCT01997125). Data were collected from a human participant with CI5 AIS A tetraplegia who had been implanted with a 96-channel Utah electrode array (Blackrock Microsystems, Inc., Salt Lake, UT, United States) in 2014. The injury occurred 4 years prior to implantation, and the data analyzed in the current experiments were collected up to 5 years post-implantation. The data analyzed for the SPC process were collected over the course of the entire 5-year dataset. These data were obtained at the beginning of each session while the study participant closed his eyes and rested for 60 s.

The datasets used for channel-masking experiments were collected over 65 sessions spanning 2.4–5 years post-implantation, and are an extension of the experiments reported by Schwemmer et al. (2018) and Skomrock et al. (2018). For the channel-masking experiments, the participant performed an imagined movement task consisting of four separate movement cues: index finger extension, index finger flexion, wrist extension, and wrist flexion. A rest period separated each of the cue presentations. A single 104-s block comprised four repetitions of each of the four different movement cues in a randomized order, for a total of 16 cued movements. Each of the 16 cues was presented in 2.5 s windows, separated by 4 s of rest. The participant did not receive any real-time feedback during the block. Two such blocks of data were collected consecutively on each day of the experiment. Both blocks of the first 30 sessions (collected between 771 and 1,130 days after implantation of the MEA) served as the training data. Both blocks of the subsequent 10 sessions (collected between 1,144 and 1,234 days after implantation) served as a validation set. The first block of the final 35 sessions (collected between 1,241 and 1,736 days after implantation) was used for the unsupervised updates, while the second block was reserved as test data. Four sessions from the training set and six sessions from the test set with known channel disruptions (as detailed in Section “Damaged Channels Identified by the Statistical Process Control Process”) were excluded to isolate the effects of simulated disruptions and better quantify the effect of the masking layer over different amounts of damage.

### Signal Processing and Simulating Disruptions

Neural signal processing was performed similarly to our previous work (Bouton et al., 2016; Colachis et al., 2018; Schwemmer et al., 2018; Skomrock et al., 2018; Bockbrader et al., 2019). Neural voltages were sampled at 30 kHz with a 0.3 Hz first-order high-pass and a 7.5 kHz third-order low-pass Butterworth analog hardware filter. This recorded signal was processed to calculate a single feature, mean wavelet power (MWP), for each channel (Friedenberg et al., 2016; Zhang M. et al., 2018). To calculate MWP, the 30-kHz data were decomposed with a ‘db4’ mother wavelet and 11 wavelet scales. Wavelet coefficients were extracted for scales 3, 4, and 5, which span a frequency range of 234–1,875 Hz. The wavelet coefficients were divided into non-overlapping 100 ms windows. The temporal average of the wavelet coefficients of each 100 ms window then was taken, resulting in a single value per channel and per wavelet scale in each window. Next, these averaged wavelet coefficients for each channel were individually standardized by subtracting out the mean and dividing by the standard deviation of each channel over a single block of data. During the training period, each block of data was standardized to itself, while during the testing period, the mean and standard deviation of the first block was used to standardize both the first and second blocks. Once the 3 × 96 = 288 features were standardized, the three averaged and standardized coefficients for each channel were then averaged together to calculate the MWP for each channel, resulting in 96 features, one for each channel for each 100-ms time bin. The MWP over a sliding window of 900 ms (or nine 100 ms time bins) was used as input to the neural network models tested model. Cues were shifted by 800 ms relative to the MWP input for accuracy calculations during both training and testing to account for reaction and system lag times. The MWP with the 800 ms cue shift was also used as input for the SVM tested, but instead of the 900 ms sliding window, a boxcar filter was used to average over the most recent 1 s of data.

To test the efficacy of the masking layer, channel damage was artificially simulated in the test data. Channels were ordered by their mutual information with the cues, and the 1, 5, 10, 15, 20, 30, and 50 channels with the highest mutual information were artificially corrupted to represent scenarios of mild to severe disruption that would present significant challenges to the decoder. Performance was also tested with no simulated corruption as a control condition to establish baseline decoder performance under ideal conditions. The sklearn package (Pedregosa et al., 2011) in Python was used to calculate mutual information between cues and channels. This package calculates mutual information in accordance with the method proposed by Ross (2014). The simulated corruption was based on true damage observed in the MEA, namely three electrically floating channels caused by Pedestal/MEA damage as discussed briefly in section “Pedestal and/or Microelectrode Array Damage Identified by Minimum Average Absolute Correlation” below and in more detail in Colachis et al. (2021). We simulated a corrupted channel as a linear combination of the raw 30 kHz signals from each of the three floating channels during a representative test session using randomly generated weights. These three channels’ signals were combined in order to create examples that were characteristic of floating channels but still variable between simulations. For each of the selected top channels on each session of the test set, an artificially corrupted channel was simulated and MWP values from the simulated data replaced the true uncorrupted MWP values. Further, to account for the variability in model performance from different random initializations of neural network parameters and different simulations of corruption, each model was trained and tested using ten different random seeds.

### Statistical Process Control Approach to Identifying Damaged Channels

Central to SPC methodology is the production of control charts, for which a numerical summary of the data is plotted over time alongside control limits that are set a predetermined number of standard deviations away from the mean of the charted statistic. For example, $\bar{X}$-charts display the means of successive samples and are useful for monitoring shifts in the data over time. *S*-charts display the standard deviations of successive samples and are useful for detecting extreme differences among the observations in a sample. Samples are classified as “out-of-control” if they meet certain pre-specified out-of-control conditions, for example if an individual point exceeds the control limits or if a run of successive points exceeds a more permissive limit. See Western Electric Company (1956) for details on different types of out-of-control conditions that are commonly used. We adapted classical SPC techniques to accommodate expected changes in neural data over time and used them to automatically detect and identify problematic channels as described below. This process requires no active input from the user; the data analyzed for this process is collected during rest periods or during impedance testing.

The disruption-identification process is comprised of three general steps: (1) transformation of the raw neural data into array-level metrics appropriate for SPC, (2) flagging of *sessions* with out-of-control signals via SPC, and (3) identification of individual problematic *channels* using the Grubbs outlier test when the array-metrics are deemed out-of-control. These steps are described further below.

#### Transformation of Raw Neural Data Into Array-Level Metrics

Four primary metrics based on both channel impedances and voltage recordings were calculated and monitored to detect signal disruptions. These metrics were selected based on their perceived association with possible types of biological, mechanical, and material damage to the MEA (see Dunlap et al., 2020) and their common usage in BCI applications. We note that the SPC approach could be used to monitor any number of other BCI metrics, although we believe the four presented here should be sufficient to identify most significant disruptions.

(1) The impedance of each channel was measured at 1 kHz using a 10 nA peak-to-peak sinusoidal current by the Blackrock Impedance Tester at the beginning of each experimental session, recording the average impedance for each channel and day.
Disruptions in the impedance data might reflect breaks in the conductor pathways or instances when the channels short to ground.

The remaining metrics were based on voltage recordings. Electrostatic discharge artifacts in the voltage data were detected using a peak prominence of 125 μV and a maximum peak width of 10 samples and replaced with a linear interpolation to reduce edge effects during filtering prior to their use in the SPC process. These artifact detection parameters were chosen based on experimentation and to avoid replacement of neural spikes. The voltage for channel *i* with electrostatic discharge artifacts removed is represented by *v* in the equations below.

(2) *V*_range_ was calculated as the difference between the maximum and minimum voltages recorded for each channel after a 250 Hz fourth-order high-pass Butterworth filter had been applied as described in Equation 1 below. This calculation differs from the standard calculation of peak-to-peak voltage, which aims to measure the quality of the action potential by taking the difference between the maximum and minimum voltages at threshold crossings rather than over the whole signal as is done for the *V*_range_ calculation. Here Butterworth_r,f_ represents the response of a Butterworth filter of order *r* and passing frequencies above *f*,

$$V_{r\,a\,n\,g\,e_{i}}=\max\left({\text{Butterworth}}_{4,250}\,\left(v_{i}\right)\right)$$

(1) $$-\text{min}\,\left({\text{Butterworth}}_{4,250}\,\left(v_{i}\right)\right)$$

*V*_range_ is expected to detect connector disruptions and sources of abnormal artifacts such as floating channels.

Finally, the voltage recordings were also used to calculate 96 × 96 matrix of pairwise correlations between channel voltages. These matrices were used to generate the two final metrics described below. The absolute value of all correlations was used in the calculation of the correlation-based metrics because the magnitude rather than the direction of the association is of interest.

(3) The maximum absolute value of all pairwise correlations between all channels *i* and *j* was determined for each day as described in Equation 2.

(2) $$M\,a\,x\,i\,m\,u\,m\,a\,b\,s\,o\,l\,u\,t\,e\,c\,o\,r\,r\,e\,l\,a\,t\,i\,o\,n=\max_{i\neq j}\left(\left|c\,o\,r\,\left(v_{i},v_{j}\right)\right|\right)$$

When two channels are electrically shorted, one channel’s signal will essentially be copied onto another, and their signals thus are expected to be abnormally highly correlated.
(4) The minimum average absolute correlation over all channels was also calculated for each day as described in Equation 3. For each channel, the mean absolute value of all pairwise correlations between the voltages of the channel of interest and each of the 95 other channels was taken. The minimum of these 96 channel-wise averages was then selected for each day.

M⁢i⁢n⁢i⁢m⁢u⁢m⁢a⁢v⁢e⁢r⁢a⁢g⁢e⁢a⁢b⁢s⁢o⁢l⁢u⁢t⁢e⁢c⁢o⁢r⁢r⁢e⁢l⁢a⁢t⁢i⁢o⁢n

(3) $$=\min_{i\in \left[1,96\right]}\left(\frac{1}{95}\,\sum_{j=1,i\neq j}^{96}\left|c\,o\,r\,\left(v_{i},v_{j}\right)\right|\right)$$

All channels are situated in a small area of the brain, and record signals that overlap to some degree. Therefore, when channels no longer adequately detect neural signals due to damage, we expect a corresponding decrease in correlation with the other channels.

The above list of metrics is not meant to be exhaustive, and additional metrics can be included in the SPC framework to reveal other types of disruptions to the recorded neural signal. However, as we will show, we believe these four metrics to be sufficient for identifying many common disruptions.

#### Flagging of Sessions With Abnormal Signals via Control Charts Based on the Metrics Calculated

Control charts were produced for each of the four metrics above to identify sessions with abnormal signal behavior. The control limits for standard control charts depend on the mean and standard deviation of the control data, and the sample size for each time point. In this application these values can change at every time point. To avoid charts with varying control limits, individual values were standardized and adjusted. At each time point the overall mean ($\bar{X}$) and within-day standard deviation (σ_w_) were calculated for the control data and each observation at the given timepoint was standardized by subtracting $\bar{X}$ and then dividing by σ_w_. The average range between consecutive timepoints in the standardized control data ($\bar{R}$) was calculated. Control limits for typical individual $\bar{X}$ charts are $\text{Target}\pm 2.66\times \bar{\text{R}}$. The target for the standardized values is 0 which means that the control limits for the daily value divided by $\bar{R}$ are constant, ±2.66. Similarly for the *S*-charts, the within-day standard deviation of the standardized values is calculated and adjusted. The control limits for these values are ±3.00. This approach was adapted from the method described by Bothe (1990). The Type I error rate for each control chart, the probability that a given datapoint appears out-of-control when the result is in fact due to random variation, is approximately 0.27% for the two-sided charts and approximately 0.13% for the one-sided charts. These values can be tuned to balance sensitivity and specificity appropriately such that the limits can be increased if lower Type I error rates are desired and decreased to lessen the risk of Type II error or if the system is failing to identify known disruptions. For the development of this method, the selection of the control limits was guided by identification of known disruptions in the data.

Classical SPC methodology assumes that in the absence of a disruption, data follow a Gaussian distribution with a constant mean and variance. However, these assumptions were not initially met for the metrics produced. For example, channel impedances were found to decay with decreasing variance over time, approximately following an exponential decay model with stable variance over the log of time. Similar properties were observed for *V*_range_. When this occurs, it is common to transform the data to better meet the assumptions required for SPC. Therefore, exponential decay models were fit to impedance and *V*_range_ metrics. Correlations between channels increased slowly over time, and thus logarithmic growth models were fit to the correlation-based metrics. Model residuals were used to construct control charts. To simplify interpretation, all control charts were standardized. That is, the control limits are kept constant for the duration of the study, and the datapoints are scaled accordingly. Specific details on the construction of control charts for each metric are given below.

(1) Both $\bar{X}$- and *S*-charts were constructed using the residual differences between the observed log-impedances and the predictions from the exponential decay model. To further allow for natural drifts in the neural signal over time, only the last 50 in-control observations were used to generate the impedance control charts.
(2) Both $\bar{X}$- and *S*-charts were constructed for the *V*_range_ exponential decay model residuals. In contrast to the impedance control charts, all in-control observations were used to generate the *V*_range_ charts.
(3) The $\bar{X}$-chart was constructed for the logarithmic growth model fit to maximum absolute correlations. All in-control observations were used to construct the chart. To avoid flagging channels that were only moderately correlated and therefore not representative of electrical shorting, only those absolute correlations that were both above the upper control limit and greater than 0.90 were flagged as out-of-control.
(4) The $\bar{X}$-chart was constructed for the logarithmic growth model fit to minimum average absolute correlations. All in-control observations were used to construct the chart.
  Note that no *S*-charts were constructed for the correlation-based control charts because only one maximum/minimum average absolute correlation is available for each day.

By design, we expect that some out-of-control signals will be false-positives. Treating all out-of-control signals as of indicative of sensor damage could therefore lead to an overly sensitive classification of damage, such that many channels that are functioning appropriately may be excluded from the model. Thus, our out-of-control condition was set such that only cases where one of the monitored metrics was outside of control limits for two or more consecutive sessions were selected for further investigation. The minimum amount of time that a metric must be out of control before being flagged for further investigation can be adjusted based on balancing tolerance for false positives and the cost of delayed identification. The use of two sessions as the required minimum for the current dataset seemed to reliably identify the known disruptions in the MEA while minimizing false positive alarms.

#### Identification of Individual Problematic Channels Using the Grubbs Test When the Array-Metrics Exceed the Control Limits

Our SPC approach flags problematic sessions based on array-level metrics. Thus, once a day has been flagged, the problematic channels still needed to be identified. To identify which channels were disrupted, the Grubbs test for outliers (Grubbs, 1950) was performed across all channels and for each control metric on each session flagged by the control charts. A significance level of 0.01 was used. The Grubbs test assesses whether the largest absolute deviation from the session mean across is significantly higher or lower than expected for each channel based on the assumption of normally distributed data. For each session, if the flagged channel is identified as an outlier, the channel is removed from the dataset and the test is performed again on the next most outlying channel. This process is repeated until no new outliers are found. Only channels that both were classified as outliers by Grubb’s test and occurred on sessions flagged on the control charts for two or more consecutive sessions were deemed “corrupted” and would be subjected to remediation. The same channels must be identified on both sessions to be considered outliers.

We emphasize that the SPC approach provides a framework for quantifying and flagging abnormal signal behavior. The specific values and transformations described above can be easily customized to comply with the needs and allowable risks of future systems while using the same SPC framework.

### Decoder Architecture and Training

#### Deep Neural Network Decoders

The deep neural network decoder introduced in Schwemmer et al. (2018) and summarized in Figure 1C was used as the base decoder. The unsupervised updating procedure also introduced by Schwemmer et al. (2018) was used to allow the neural network to adapt to changes in neural output over time without requiring explicit retraining or collection of new labeled training data from the user. This procedure is related to semi-supervised learning, where the models own predictions can serve as pseudo-labels to augment the training dataset (Rosenberg et al., 2005). During the unsupervised updating paradigm, for a given day of test data, the model from the previous day was used to predict the movement cues for the first block of data. These predictions were concatenated with the previous ten training or updating blocks and used as pseudo-labels to update the model weights. This differs slightly from the procedure used by Schwemmer et al. (2018), where all the training data and pseudo-labels generated up through the test day were concatenated and used for the update procedure.

Additional data augmentation and regularization techniques were also implemented to improve generalizability of the model. The mixup algorithm (Zhang H. et al., 2018), which creates additional synthetic training data using linear combinations of the training features and cues, was applied during model-fitting. Data augmentation using mixup is thought to reduce overfitting and increase robustness of the model. Dropout of a randomly selected 50% of the forward connection weights in each layer was also implemented during each epoch to further prevent overfitting to the training data. To remain consistent with the architecture used by Schwemmer et al. (2018), the default pytorch LSTM was modified to apply dropout to the recurrent connections of the network as well as to the forward connections. The recurrent dropout percentage was set to 25% for all models.

Categorical cross-entropy loss was used during the initial training period when true cue labels were available. However, to compensate for the uncertainty in the true cue labels during the use of the unsupervised updates, the bi-tempered logistic loss function was used during the update period (Amid et al., 2019). Temperature parameters *t_1_* = 0.7 and *t_2_* = 1.3 were used, leading to a non-convex function that produces bounded, heavy-tailed losses. This loss function offers more flexibility than the modified cross-entropy function (Reed et al., 2014) which was used by Schwemmer et al. (2018) for the unsupervised updating procedure. The new loss function was chosen based on experimentation suggesting it led to improved performance, possibly due to a lower sensitivity to outliers (Amid et al., 2019).

The 1cycle policy (Smith, 2018) was used during training to promote model convergence in a relatively small number of epochs via methodical selection of the following model fitting hyperparameters: learning-rate, momentum, weight decay and batch size. This is a well-established approach to for selecting neural network hyperparameters that is becoming standard in deep learning applications (Howard and Gugger, 2020). An early stopping criterion was also applied to determine the number of epochs that should be used for performing both the initial training and the unsupervised updates. If the decoder validation accuracy did not change by more than 0.01 for two epochs, the update process was halted. These techniques both promoted efficiency in training and helped to standardize comparisons in training time.

Table 1 summarizes the decoder training details in terms of the datasets used, disruptions simulated, and fitting techniques applied. The model was coded and trained in python 3.7.3 using pytorch 1.1.0 module (Adam et al., 2019) with the fastai 1.0.57 framework (Howard and Gugger, 2020).

**TABLE 1:** Dataset used for all experiments and training parameters common for all DNN models.

Neural feature:	Mean wavelet power (MWP)
Number of blocks for test period:	30 blocks
Number of blocks for initial training:	60 blocks
Number of blocks for unsupervised updating:	11 blocks (10 historical + 1 current)
Number of epochs:	Determined by early stopping (patience = 2, accuracy delta = 0.01, evaluation blocks = 20)
Data augmentation:	Mixup during update loop
Dropout:	0.50 Forward Layers 0.25 Recurrent Layers
Loss function:	Categorical Cross-Entropy Loss during initial training, BiTempered Loss during unsupervised updates
Channels corrupted:	The [0, 1, 5, 10, 15, 20, 30, 50] most important channels
Simulated corruptions:	Random linear combinations of floating channel data
Damage introduced:	First day of test period

#### Support Vector Machine

For comparison, we also fit a daily-retrained SVM with non-linear Gaussian radial basis function kernels with a γ parameter value of 0.005. This value was selected based on previous experiments (Bouton et al., 2016; Sharma et al., 2016). The sci-kit learn toolbox (version 0.22; Pedregosa et al., 2011) in Python was used to train the SVM. Channel masking was not applied to the SVM. Rather, damaged channels were omitted from the input manually to imitate current practices and as a best-case representation of daily retraining.

### Channel Masking Layer

The channel masking layer is initialized as an identity layer with no added bias. It is inserted as a non-trainable first layer in the neural network architecture and its output is fed directly into the neural network decoder. The weights along the diagonal will be set to either 1, to pass input from “healthy” channels on to the decoder, or to 0, to exclude damaged channels to the model. The determination of which channels to exclude could be done manually, or the outliers identified by the SPC algorithm could be used to determine which weights get set to zero on a given day. If no weights are set to zero, the masking layer remains an identity layer and has no effect on the decoder.

### Decoding Approaches in the Presence of Damaged Channels

Four different methods of adjusting to damaged channels were compared. These methods included:

(1) The unsupervised neural network model (uNN) based on Schwemmer et al. (2018) trained using all channel inputs over the training period and given unsupervised updates for each test session with no explicit adjustments made to accommodate the damaged channels (uNN-NOMASK). This model was tested as a baseline condition.
(2) The uNN framework trained using all channel inputs over the training period and given unsupervised updates on each test session with the masking layer inserted immediately prior to the decoder architecture to zero out the corrupted channels (uNN-MASK). The decoder architecture was identical to that of the uNN-NOMASK except for the addition of the masking layer.
(3) The uNN model retrained from scratch from the beginning of the training period and given unsupervised updates on each test session with the damaged channels removed from the dataset (uNN-RETRAIN). The decoder architecture was identical to that of the uNN-NOMASK except the initial layer was modified to accommodate the reduced number of channels. For example, in the case where the top 10 most important channels were artificially corrupted, the model would take an 86 × 9 dimensional array as its input instead of the regular 96 × 9 array. This method is the most intensive of those tested in terms of data storage requirements and retraining time.
(4) A SVM trained from scratch using only labeled data from the first block on the day of the test and with the damaged channels removed from the dataset (SVM-REMOVE). The inputs to the SVM model are identical to those to the uNN models, with the exception that a 96 × 1 array representing a boxcar-filtered average 1 s of data is fed to the model at each time point instead of the 96 × 9 time series array that is used for the uNN models. This model is trained as an additional baseline, as it is similar to approaches used in current laboratory-based systems (Fernández-Delgado et al., 2014; Bouton et al., 2016; Sharma et al., 2016; Colachis et al., 2018).

To assess the effect of unsupervised updates, the uNN-MASK and uNN-RETRAIN approaches were also tested both without updates applied and with supervised updates using ground-truth labeled data.

### Definition of Key Metrics

Two quantitative metrics referred to as accuracy and success were used to assess the decoder performance. Accuracy measures the ability of the decoder to match cued movements at a specific time and represents the standard machine learning classification definition of accuracy, while success measures predictive ability over the full cued movement period and approximates how an observer might score each cue as a binary success or a failure. Specifically, accuracy was calculated as the percentage of time bins for which the decoder prediction matched the cued movement or rest after the cues had been shifted by 800 ms to account for lags in reaction time. The success rate was calculated as the percentage of cues where the correct movement was predicted during the cue window and sustained for at least 1 s of the 2.5 s cued movement period. Correct predictions did not need to be consecutive to count as a success, and the predicted movement was allowed to extend into the following rest period for up to 0.9 s. Unlike for accuracy calculations, prediction of rest periods was not factored into the calculation of success rates.

Concretely, the accuracy for a 104-s block would be the percentage of the 1,040 100-ms time bins for which the decoder prediction matched the cue and the success rate would be the percentage of the 16 cued movements that were predicted correctly for at least one (non-consecutive) second.

### Quantitative and Statistical Analysis

To test for differences between modeling approaches, mixed effects models were fit separately to the accuracies and successes calculated over each test session. They included fixed effects of the number of channels affected, decoder type, and the interaction between number of channels and decoder type, and a random effect of random seed. All effects were modeled as categorical variables. The Holm method of adjustment for multiple comparisons was used to maintain a family-wise error rate of 0.05 over all stated comparisons between models and numbers of channels affected within a performance metric (Holm, 1979).

The computational requirements to train each neural network model were quantified as the number of equal-sized batches necessary to satisfy an early stopping criterion, such that the training and update processes were halted if the validation accuracy did not improve by more than 0.01 for two epochs. The data storage requirements are measured as the number of blocks required to train and update the model.

## Results

The following sections discuss results from the modified SPC process applied over the 5-year dataset and from the channel masking experiments with simulated disruptions. The SPC results compare known disruptions that were manually identified over the course of the study to the automatic detection of disruptions by our SPC algorithm. Then, the channel masking simulation results compare decoder performance and associated computational requirements across the various approaches for addressing disrupted channels.

### Damaged Channels Identified by Statistical Process Control

**FIGURE 2:**
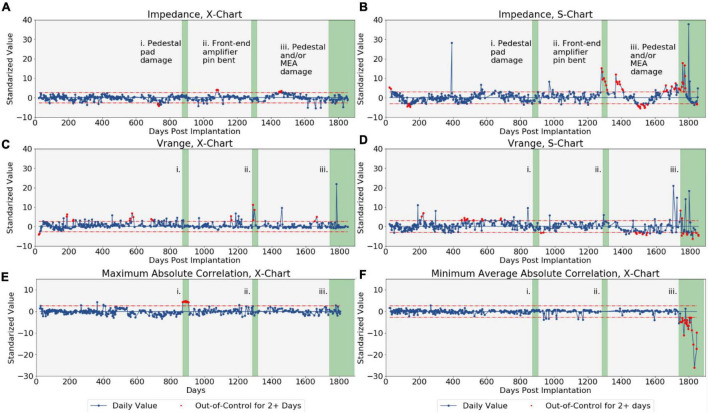
The control charts produced for each of the four array-level metrics monitored by the statistical process control-based algorithm. Both $\bar{X}$ and S-charts were produced for impedance and voltage range, while only $\bar{X}$-charts were produced for the correlation metrics. Dotted red lines represent control limits. The red points indicate metrics flagged for having been out-of-control for at least two consecutive sessions. The green regions represent periods of known damage. (A) Impedance $\bar{X}$-chart. (B) Impedance S-chart. (C) *V*_range_ $\bar{X}$-chart. (D) *V*_range_ *S*-chart. (E) Maximum absolute correlation $\bar{X}$-chart. (F) Minimum average absolute correlation $\bar{X}$-chart.

Our statistical process control method was able to clearly identify several real-world disruptions due to various types of failures in the signal collection chain. Control charts were constructed for the impedance, *V*_range_, maximum absolute correlation, and minimum average absolute correlation metrics over the study period, and 148 sessions were flagged as out-of-control (Figure 2, out-of-control sessions highlighted in red). After applying the Grubbs test, 100 instances of outlying channels were identified from these out-of-control signals and so were classified as disruptions by the modified SPC approach. Of these 100 suspected disruptions over the 387 sessions examined (with 96 channels tested each day, for a total of over 37,000 channels examined), 79 were associated with known instances of damage. The ability of SPC to correctly identify these known disruptions helps to generate confidence that the method can detect the disruptions it was designed to detect. In the following, we note three examples of out-of-control observations identified by SPC with clear links to known disruptions in electrode array channels. A detailed discussion of observed disruptions to the neural signal for the duration of this study is available by Colachis et al. (2021).

#### Pedestal Pad Damage Identified by Maximum Absolute Correlation

Electrodes 78 and 87 had been shorted together by a scrap electrode pad that had fallen loose while the patient cable was being connected to the electrode array pedestal near day 871. This electrical short was detected on the $\bar{X}$-chart for maximum absolute channel correlation of our SPC process for 7 out of the 10 sessions between days 871 and 906 after implantation, when abnormally high and out-of-control correlations were observed for these two channels (see Figure 2E, region i). The electrical short effectively duplicated the signals across two channels and therefore the subtle effect was only apparent by observing the abnormally high correlation between the two channels. The correlation of these channels returned to being in-control after repairs of the system were made.

#### Front-End Amplifier Pin Bend Identified by Impedance S-Chart

A front-end amplifier pin on the system had been bent near day 1,283, likely due to repeated connections and disconnections of the patient cable, which caused disruptions in Channel 1 of the MEA. This disruption was detected by our SPC process as out-of-control observations (between approximately 4 and 15 standard deviations about the mean) on the impedance S-chart (see Figures 1, 2B, region ii) and subsequent identification of Channel 1 as an outlier by the Grubbs test for all seven sessions between days 1,283 and 1,316. Channel 1 had also been identified as an outlier for three of the seven sessions on the *V*_range_ $\bar{X}$-chart. After repair of the damaged pin, the Channel 1 impedances returned to in-control values.

#### Pedestal and/or Microelectrode Array Damage Identified by Minimum Average Absolute Correlation

Channels 85, 93, and 96 are believed have become electrically floating from day 1,742 onward, likely due to material degradation to the pedestal and/or MEA from long-term use. These floating channels record surrounding noise rather than neural activity, which manifests in the data as abnormally low correlations with surrounding non-floating channels and high correlations amongst the floating channels. Consequently, both the *S*-chart for *V*_range_ and the $\bar{X}$-chart for minimum average channel correlations all had out-of-control observations from day 1,742 after MEA implantation onward (see Figures 1, 2F, region iii). Channels 85, 93, and 96 were identified as outliers for minimum average absolute correlation by the Grubb’s test during this period. Of the 25 sessions after day 1,742 disruptions were identified in Channel 85 for 21 sessions, Channel 93 for 19 sessions, and Channel 96 for 22 sessions. The floating channels cannot be repaired without surgical intervention.

The modified SPC approach presented here was able to successfully identify three known occurrences of damage to the MEA channels. The control charts for each of the four monitored metrics flagged at least one of these cases of disruption, providing evidence that each of impedance, voltage, and channel voltage correlations is informative for detecting disruptions that may occur in implanted Utah arrays.

### Model Performance With Simulated Damage

**FIGURE 3:**
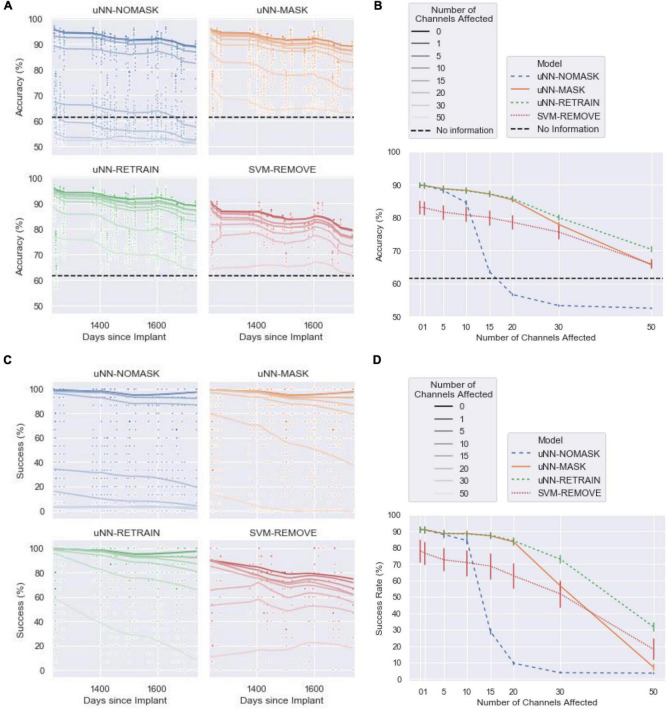
The performance of the decoders with increasing numbers of channels affected by corruption. Simulated damaged was introduced on the first day of the test set. The uNN-NOMASK, uNN-MASK, and uNN-RETRAIN were tested over 10 different random initializations, while only one SVM was tested for each day and number of channels affected. (A) The accuracy over the 1.4-year test period. (B) The mean accuracy across 100-ms time bins as a function of the number of channels dropped. Error bars represent 95% confidence intervals. (C) The success rate over for each 2.5 s cue over the 1.4-year test period. (D) The mean success rate across 100-ms time bins as a function of the number of channels dropped. Error bars represent 95% confidence intervals.

**FIGURE 4:**
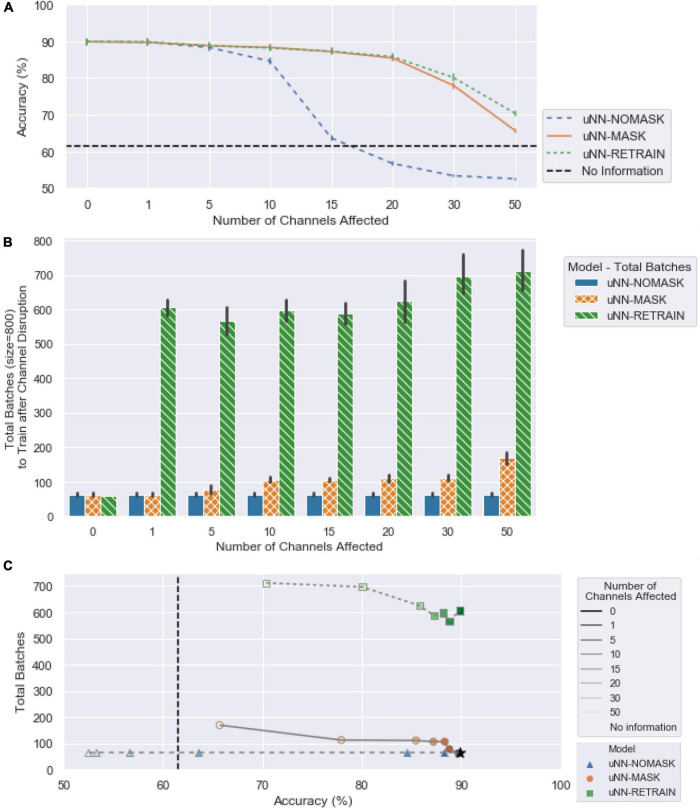
(A) The mean accuracy for the three uNN decoders, replotted from Figure 3B with *x*-axis ticks realigned to match (B). (B) The computational requirements of the uNN decoders as measured by the number of batches required for training after disruptions are introduced. When zero channels are affected, no masking or retraining takes place, and the model would only receive unsupervised updates. The number of batches required is according to an early stopping criterion and is averaged over each of the 10 random initializations applied. Error bars represent 95% confidence intervals. (C) The number of batches required as a function of decoder accuracy.

The following sections compare the effectiveness of the tested approaches in addressing increasing amounts of simulated damage. Accuracy and success of each of the four models are presented (see Figure 3) as well as their retraining time burden (Figure 4). Note that an accuracy of 61.5% is equivalent to the decoder predicting rest for all cues.

#### uNN-NOMASK and Support Vector Machine Performance Degrade With Increasing Amounts of Damage

We first compare performance of the uNN with no explicit handling of damaged channels (uNN-NOMASK) to the daily-retrained SVM (SVM-REMOVE) that removes the damaged channels from the input. The uNN-based decoders (which were all equivalent when no corruption was introduced) achieved superior performance to the SVM in the baseline scenario when no channels were disrupted. With no simulated damage, the uNN decoders achieved a mean accuracy of 89.87 ±6.97% (mean±standard deviation) and a success rate of 90.97 ±15.61% over the test period. The SVM-REMOVE achieved a lower mean accuracy of 83.20 ±6.23% and a success rate of 77.90 ±6.23%.

The low relative performance of the SVM-REMOVE continued through the disruption of the 10 most important channels, such that it was the poorest-performing model in these cases. The performance of the uNN-NOMASK decreased drastically after 15 or more channels were affected, however, and fell far below the performance of the other models. The performance of the uNN-NOMASK was near or below chance levels when 15 or more of the most important channels are affected.

The uNN-NOMASK required an average of 63 batches for the daily unsupervised updating procedure regardless of the amount of simulated damage introduced. A total of 31 blocks of data were used, 11 of which were used for the daily update procedure and 20 for the validation dataset. The daily-retrained SVM would only require one block of data stored at a time and can be trained in fraction of the time of a deep learning model, but importantly, would require the user to actively spend time each day collecting labeled data which is not required for the uNN models.

These results demonstrate that a uNN approach is superior to the SVM approach in most use cases when no or minimal array damage is present, both in terms of accuracy and in terms of the computational and time burdens on the user. When greater amounts of damage are present in the array, additional means of addressing the damage are necessary for the uNN to be viable.

#### Masking Allows uNN to Maintain High Performance Despite Damage

Both uNN-based models that explicitly handle the corrupted channels maintained high performance through a considerable amount of corruption introduced to the neural signal. Unlike for the uNN-NOMASK and SVM-REMOVE, mean accuracies remained above 85% and mean success rates remained above 80% for both the uNN-RETRAIN and the uNN-MASK for corruption of up to the 20 most important channels.

When 10 or more channels were disrupted, masking the corrupted channels significantly improved accuracy [increase of 3.75 ±3.07%, *t*(8639) = −7.51, *p* < 0.0001 for 10 channels disrupted] and success [increase of 4.36 ±7.20%, *t*(8639) = −3.28, *p* = 0.0195 for 10 channels disrupted] when compared to the no masking. The benefit from channel masking was massive when damage was simulated in 20 channels (mean difference in accuracy: 28.75 ±6.67%; mean difference in success: 73.89 ±19.72%).

Minimal additional data is required to adjust the uNN with the channel masking layer in place, as the uNN-MASK only utilizes data used in the daily unsupervised updating procedure. The computational times for the uNN-MASK and uNN-NOMASK are simply those needed for the unsupervised updates according to the early stopping criterion. The uNN MASK required more batches to update than the uNN-NOMASK due to the increased need to recalculate weights as more channels are masked. The uNN-MASK required nearly three times as many batches as the uNN-NOMASK to update when 50 channels were affected, but otherwise required fewer than twice as many batches.

#### uNN-RETRAIN Provides Marginal Improvements Over Masking

For all cases in which simulated damage was introduced into the data, the uNN-RETRAIN attained accuracies and successes that were superior to or not statistically different from all other models. The high relative performance of this model is unsurprising, as it supplies the decoder with the largest amount of labeled data for use in adapting to the loss of the affected channels.

However, retraining the uNN from scratch only yields statistically better accuracy and success compared to masking in instances of greater damage, when 30 or 50 of the 96 channels are affected. When 30 channels are affected, the accuracy benefit for the uNN-RETRAIN over the uNN-MASK is 2.14±5.04% [*t*(8639) = −4.28, *p* = 0.0003] and the success benefit is 16.29 ±19.02% [*t*(8639) = −12.26, *p* < 0.0001]. When 50 channels were disrupted, the average benefits in accuracy and success obtained by retraining from scratch over masking were 4.69 ±3.96% and 24.55 ±17.03%, respectively. Despite these benefits, performance was still relatively poor for the uNN-RETRAIN with the 30 or 50 most important channels disrupted, when accuracies were at most 80.06% (8.6%) and success rates were at most 72.99% (22.48%).

Retraining the model from scratch while omitting the damaged channels comes at the cost of processing time, as it can take between approximately 4 (for 50 channels removed) and 10 (for one channel removed) times longer on average than simply performing the unsupervised update after masking. In contrast to the 31 blocks used to implement the uNN-MASK, a total of 81 blocks were used to retrain the model from scratch. This included all 60 blocks of the labeled data from the training period in addition to the 20 blocks of validation data. As the test period continued, 11 blocks used for daily updates were also added to the data stored.

#### Unsupervised Updates Are Essential to Masking Performance

The intended purpose of unsupervised updating is to allow the algorithm to adapt to gradual changes in the neural signal over time. However, the updating process is also critical for the success of the channel masking procedure. The maximum masking benefit for a model that did not receive unsupervised updates was only 15.2 ±5.00% in accuracy and 44.61 ±17.11% in success, which occurred when 15 corrupted channels were masked. In contrast, when unsupervised updating was performed the accuracy and success increase by 23.57 ±5.68% and 58.19 ±17.78%, respectively, for 15 channels affected. The model that masked corrupted channels also performed statistically significantly worse than the model that was retrained from scratch when updates were not performed for either model after only 10 channels were affected [accuracy: 2.17 ±2.44%, *t*(8639) = −3.78, *p* = 0.0006; success: 4.86 ±9.44%, *t*(8639) = −3.52, *p* = 0.0017], compared to 30 channels affected when updates are performed. The unsupervised updating procedure therefore is necessary to readjust the weights after the most important channels have been omitted, and thus those which the decoder had likely been most reliant on for information, had been dropped.

## Discussion

Neural sensors are vulnerable to damage over time (Barrese et al., 2013; Dunlap et al., 2020; Colachis et al., 2021), and this vulnerability will increase as BCIs become used in more unpredictable environments outside the laboratory. Identifying and adapting to these disruptions will thus be essential in a deployed BCI system. Here, we present a three-staged approach to maintaining high deep neural network performance in the presence of acute array damage with minimal computational requirements and no required user intervention. This approach includes (1) a statistical process control-based methodology for automatically identifying channels with disrupted signals, (2) a masking layer preceding the network architecture to remove input from the disrupted channels without retraining from scratch, and (3) an unsupervised updating procedure to adjust model weights to the missing information without explicit recalibration. The implementation is described here for a 96-channel Utah array, but the system can be easily extended for use with other MEAs or even other types of physiological signals, such as EMG, ECoG, or EEG data.

Traditional statistical process control techniques were adapted to identify several outlying signals that may correspond to damage to the neural sensor itself or to downstream components. The benefits of this automated damage-identification algorithm are twofold. First, the SPC procedure can alert the user or technician when a potential issue occurs, expediting repairs and triggering algorithmic adjustments. This benefit exists independently of how well the decoder adapts to the damaged channels. Second, automated identification of problematic channels enables automated compensation in the decoding algorithms, with the methods introduced here as one example. The SPC procedure may also be extended for multiple sensor arrays. Individual channel metrics like impedance would follow the same SPC approach as a single array whereas metrics that incorporate data from more than one channel like correlations could optionally be calculated across multiple arrays. Additionally, metrics that specifically compare signal quality between multiple arrays could be added to the procedure.

The conventional approach to addressing disrupted array signals is to retrain the decoder from scratch with the damaged channels removed. However, this approach requires significant time from the participant to collect new training data followed by computation time to retrain the decoder model before the decoder is usable. Furthermore, if the decoder uses historical data, that data needs to be stored and accessible for retraining the model. Our approach substantially lowers both the computational and storage burden compared to entirely retraining the model. The time and data storage requirements for the SPC approach to detect disrupted electrode signals are negligible. This scheme only requires that up to two values (one corresponding to each the X^–^- and potentially the S-charts) be stored for each of the four metrics per day. The approach shown here only requires a small amount of rest data collected each day and only a few simple calculations. However, a similar approach could be used for more frequent checks during daily use of the system to identify disruptions closer to their time of occurrence. The SPC approach could also be modified in future use cases to monitor and identify disruptions during active use of the decoder.

Once identified, masking of channels with simulated disruption effectively preserved the high performance of the decoder in the presence of moderate amounts of damage. The overall success rate of the uNN-MASK is only a few percentage points lower than in the case of no damage when up to fifteen of the most important channels in the sensor are masked. Even when 20 of the most important channels are masked, the uNN-MASK success rates remain above 80%. This is a lower success rate compared to the model’s typical performance but would still satisfy approximately three quarters of potential BCI users with spinal cord injury surveyed (Huggins et al., 2015). Without masking, the model successfully responds to cues less than a third of the time when 15 channels are corrupted. When damage was simulated in 20 channels, the uNN-NOMASK was no better than chance.

Importantly, it is also observed that the benefit obtained from channel masking is immediate, such that performance of the uNN-MASK is near the performance of the uNN-RETRAIN on the first day after the channel masking is used. Benefit from the masking layer is the most critical within the period immediately after neural signal disruptions are discovered because many cases of damage would ideally be repaired quickly after detection. Interestingly, even when labeled data is used to update the model in a supervised fashion for the first several days after damage is detected, performance is higher with channel masking turned on versus left off. This further highlights the benefits of explicitly masking damaged channels, as corrupted channels may not always flatline, but instead could have widely varying values that may cause issues for many decoders (Colachis et al., 2021).

The above results also add to the evidence that our unsupervised updating approach for neural networks has significant benefits for sustaining the robust performance requirements of BCI users while maintaining a light computational footprint that is compatible with the low-power devices that will deploy these algorithms. The benefit attained by masking substantially increased when masking was accompanied by unsupervised updates to readjust model weights. Furthermore, after damage is repaired, the previously masked channels will need to be reintroduced back into the model. The reintroduction is as simple as changing the weights in the identity layer for the relevant channels from 0 to 1. However, the decoder will have adjusted its weights to ignore input from the previously masked channels. Therefore, the unsupervised updating procedure will again be critical for readjusting the weights to reincorporate the repaired channels.

By leveraging previously learned weights that already encode much of the necessary information for decoding, we have shown that we can efficiently compensate for the disruption without compromising performance. Furthermore, Kao et al. (2017) argued that the past behavior of the neural signal may beneficially inform predictions even when the neurons recorded in previous data are no longer active. The performance of their hysteresis neural dynamical filter (HNDF) that incorporated neural dynamics from historical data with more neurons available was greater than that the performance of the NDF that was trained only on data from the day of the test. The model and architecture used in the current study are distinct from the HDNF filters used (Kao et al., 2017), but the uNN weights are informed by 2 years of historical data prior to the test period.

Additional robustness to changes in the neural signal was also incorporated during the training stage through the use of dropout and mixup. Dropout simulates loss of a random set of connection weights in each layer in order to regularize the model and prevent over-reliance on a given set of connections and is commonly used in neural network training. Mixup, which augments the training dataset by generating linear combinations of training examples, is a newer technique and we believe this is the first time it has been used in neural decoding. However, the poor performance of the uNN-NOMASK for more than 10 channels affected shows that these training strategies alone are not sufficient to maintain high performance of the BCI.

Adjustments to the model weights to accommodate the masked channels occur implicitly through the daily unsupervised updating that already takes place in the uNN model, and thus entail minimal computational requirements on top of the regular start-up procedure. Deep neural networks with unsupervised updates can perform well over time with a fraction of the training sessions used here (Schwemmer et al., 2018), which would result in more similar computational requirements for the retraining and masking approaches. However, considering the near-equivalence of the uNN-RETRAIN and the uNN-MASK when damage was simulated in up to 20 of the most important channels, the performance benefits from retraining are expected to be minimal when the decoder is retrained with only a small number of trials. Furthermore, the entire process of identifying and masking disrupted channels requires no explicit input from either the user or a technician and is thus aligned with user preferences that no intervention is required after the initial training period (Huggins et al., 2011, 2015; Collinger et al., 2013a).

### Limitations

The SPC procedure was designed to detect known disruptions in the specific Utah array under study. The settings chosen for the proposed SPC algorithm therefore may overfit to the instances of damage observed, and may need to be adapted for optimal performance on a new system. The general framework involving collecting rest data, creating features from this data, transforming the features to fit normal distribution and constant variance assumptions, setting control limits, and performing Grubbs tests for outliers is applicable across all types of electrode arrays. However, details such as the optimal features chosen, transformations applied, number of past sessions to include when fitting models, the control limits, allowable number of out-of-control sessions, and significance level of the outlier test choices were made to tune the SPC algorithm to detect the types of disruptions that the current system was susceptible to, and thus which might reasonably be expected in similar systems. These details may therefore change based on the recording modality and preferred tradeoffs between false positives and false negatives in disruption detection.

A limitation of the SPC procedure is the tradeoff between the time to identify a damaged channel and the specificity of the detection process. In the proposed system, a channel must be classified as an outlier for two sessions in a row before it is considered corrupted and further action is taken, which may correspond to several days of damage. While rest data can be collected and the SPC procedure run several times a day in a real-world scenario to reduce the time to detect disruptions, the BCI user may still be performing sub-optimally for some time before the channel masking is activated. Furthermore, if the BCI under the proposed scheme performs unsupervised updates using disrupted data during this time, performance decrements may result even after the channel damage is addressed. Alternatively, examining the input data for outlying channels after just one instance of an out-of-control metric may lead to misclassification and therefore masking of healthy channels. The experiments discussed above were run under ideal conditions, where the simulated damage was perfectly “detected” and addressed as soon as it occurred. However, the tradeoff between the time to detect a defective channel and possible information loss from masking healthy channels will need to be considered for a real-world scenario where damaged channels must be detected for an actively used system.

An important caveat of the proposed system to identify and correct for array damage is that it is only appropriate for acutely occurring damage that has lasting effects on the signal. Because the SPC procedure incorporates a waiting period before enough data is collected to trigger the channel masking and subsequent weight update, significant changes to the time scale of the detection process would be required for the system to address transient changes that may resolve themselves within minutes or hours (for example, see Dunlap et al., 2020; Colachis et al., 2021). Further, gradual decays in the neural signal may not lead to identification of outlying sessions or channels that would trigger the masking mechanism. Additional algorithmic strategies, such as the proposed unsupervised updating scheme, the use of robust features, dropout, and data augmentation are better suited to address slow changes in the neural signal over time.

## Conclusion

In BCIs that are viable for long-term daily usage, the decoders must be able to adapt to moderate channel disruptions without requiring immediate intervention from a technician. Furthermore, the solution to the disruptions should be compatible with limitations imposed in a take-home environment, including limited available data storage and hardware, minimal available retraining time, and the strong preference of users to not regularly collect new training data. Decoder models following an unsupervised updating schedule have been shown to be robust to damage to the most important channels in the electrode array, provided that the affected electrodes are explicitly identified. A modified set of statistical process control techniques can automatically identify electrodes affected by various types of acute disruptions with minimal computation and data storage. Following the identification, a masking layer prior to the full decoder architecture can remove input from damaged channels and unsupervised updates can be used to adjust decoder weights accordingly. This allows the decoder to maintain performance comparable to if the decoder were retrained from scratch, but with fewer data storage and retraining time requirements. By incorporating techniques to increase BCI decoder robustness to expected variability and abnormal signal disruptions, we can facilitate the long-term daily use of such systems.

## Data Availability Statement

The data analyzed in this study is subject to the following licenses/restrictions: data used in this study can be made available to qualified individuals for collaboration provided that a written agreement is executed in advance between Battelle Memorial Institute and the requester’s affiliated institution. Requests to access these datasets should be directed to DF, friedenbergd@battelle.org.

## Ethics Statement

The studies involving a human participant were reviewed and approved by the United States Food and Drug Administration (FDA) under an Investigational Device Exemption (IDE) and The Ohio State University Wexner Medical Center Institution Review Board (Columbus, Ohio) and registered on ClinicalTrials.gov (Identifier NCT01997125). The patient/participant provided their written informed consent to participate in this study. Written informed consent was obtained from the individual for the publication of any potentially identifiable images or data included in this article.

## Author Contributions

JV and DF conceptualized the manuscript. JV wrote the first draft of the manuscript and developed the channel masking algorithm. LA developed the statistical process control methodology with input from SC and CD. ST contributed to its implementation. All authors contributed to manuscript revision, read, and approved the submitted version.

## Conflict of Interest

The authors declare that the research was conducted in the absence of any commercial or financial relationships that could be construed as a potential conflict of interest.

## Publisher’s Note

All claims expressed in this article are solely those of the authors and do not necessarily represent those of their affiliated organizations, or those of the publisher, the editors and the reviewers. Any product that may be evaluated in this article, or claim that may be made by its manufacturer, is not guaranteed or endorsed by the publisher.
